# Supplementary material for: Early Archean origin of heterodimeric Photosystem I
Source: Heliyon. 2018 Mar 6;4(3):e00548. doi: 10.1016/j.heliyon.2018.e00548 (PMC5857716; doi:10.1016/j.heliyon.2018.e00548)
Supplement: mmc2 [file mmc2.docx]

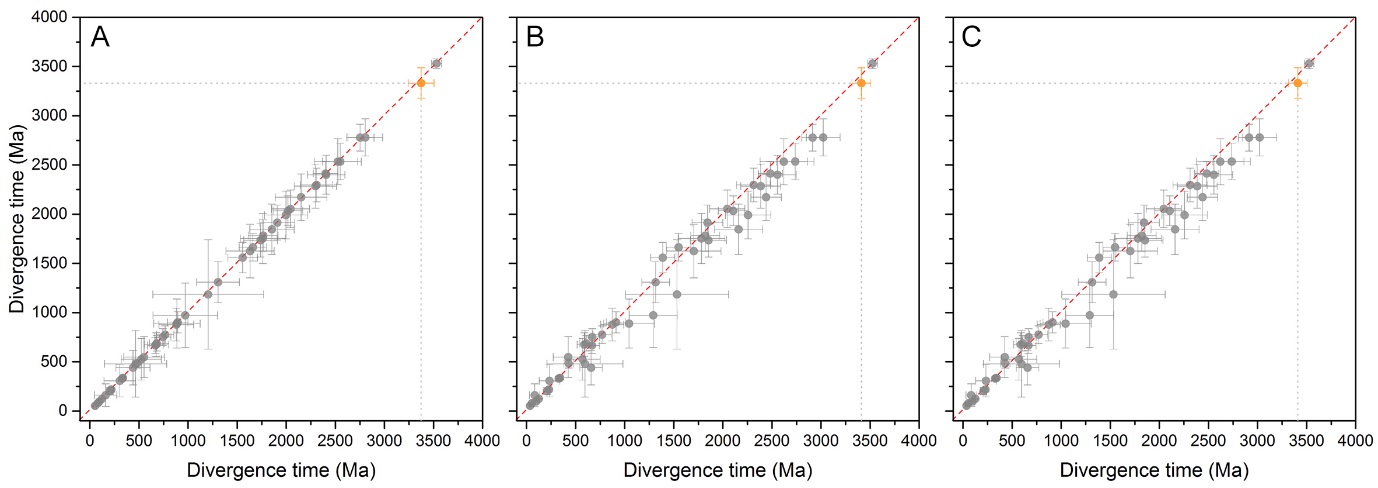


**Supplementary Figure 1.** Comparison of divergence time estimates computed using just the antenna or the core domains of the reaction centre protein. (A) Comparison of divergence times of the core domain (*x* axis) and the antenna domain (*y* axis). The molecular clock were calculated using the same parameters as the tree shown in Figure 3. That is assuming a root of 3.5 Ga and using all calibration points listed in Table 2. No difference in the estimated times were obtained when the antenna or the core domains only were used in the analysis. Comparison of the estimated divergence times of molecular clocks calculated using the full sequence of the reaction centre proteins (*x* axis) and only using the core or the antenna domain (*y* axis) are shown in panels (B) and (C) respectively. A very small deviation in the estimated times is seen with a few nodes showing slightly older ages when just the core or antenna domain was used to compute the tree. In either case the timing for the duplication of PsaA and PsaB still remained as the oldest node after the root.
